# The effects of traditional Chinese mind-body exercise on pulmonary rehabilitation in patients with chronic obstructive pulmonary disease: a systematic review and meta-analysis

**DOI:** 10.3389/fmed.2026.1740693

**Published:** 2026-02-11

**Authors:** Yuqiong Xiang, Jiangbo Liu, Zhen Zhu, Xingyu Hu, Wei Li

**Affiliations:** 1 School of Rehabilitation Medicine, Gannan Medical University, Ganzhou, Jiangxi, China; 2 Department of Orthopedics, Jiujiang Hospital of Traditional Chinese Medicine, Jiujiang, Jiangxi, China

**Keywords:** baduanjin, chronic obstructive pulmonary disease, liuzijue, pulmonary rehabilitation, tai chi, traditional Chinese mind-body exercises, yijinjing

## Abstract

**Background:**

Chronic obstructive pulmonary disease (COPD) remains the leading cause of illness and death. Traditional Chinese mind-body exercises (TCMBEs), such as Tai Chi, Baduanjin, Liuzijue, and Yijin Jing, have emerged as additional treatment options for pulmonary rehabilitation (PR). This meta-analysis sought to find out the extent to which TCMBEs affect lung function, exercise capacity, and health-related quality of life (HRQoL) in patients with COPD.

**Methods:**

Five international databases (PubMed, Embase, Web of Science, Cochrane Library, and Scopus) were examined until October 2025. Twenty-two randomized controlled trials (RCTs) with 1,871 individuals fulfilled the qualifying criteria. Outcomes included forced expiratory volume in 1 s (FEV_1_), forced vital capacity (FVC), the FEV_1_/FVC ratio, percent predicted FEV_1_ (FEV_1_ % predicted), six-min walk distance (6MWD), and patient-reported measures such as the St. George's Respiratory Questionnaire (SGRQ), COPD Assessment Test (CAT), and the modified Medical Research Council (mMRC) dyspnea scale. Data were synthesized using a random-effects model, with subgroup analyses conducted according to exercise type. The risk of bias was evaluated using the Cochrane Risk of Bias 2.0 tool.

**Results:**

Compared with usual care or conventional PR, TCMBEs significantly improved FEV_1_ (*MD* = 0.20 L; 95% CI 0.09–0.30), FVC (*MD* = 0.22 L; 95% CI 0.06–0.38), FEV_1_/FVC (MD = 3.42 %; 95% CI 2.54–4.29), FEV_1_ % predicted (*MD* = 4.98 %; 95% CI 2.53–7.42), and 6MWD (*MD* = 42.05 m; 95% CI 29.06–55.05; *p* < 0.01). Quality-of-life scores improved significantly, as reflected by reductions in SGRQ (−13.76 points), CAT (−2.62 points), and mMRC (−0.50 points). Subgroup analyses revealed that Liuzijue and Yijinjing produced the greatest gains in pulmonary function, while Tai Chi yielded the most pronounced improvement in functional endurance. No serious adverse events were reported across the included studies.

**Conclusions:**

TCMBEs are effective and safe adjuncts for COPD rehabilitation. By focusing on controlled breathing, gentle movement, and mindful awareness, these practices can enhance lung function, improve exercise capacity, and support psychological well being. Incorporating approaches such as Liuzijue, Tai Chi, and Yijinjing into structured PR programs may provide a sustainable and cost-effective way to promote functional recovery and improve overall quality of life in people with COPD.

## Introduction

Chronic obstructive pulmonary disease (COPD) is a major global health threat. It ranks as the third leading cause of death worldwide and contributes significantly to the overall disease and healthcare burden. (1, 2). COPD is marked by persistent and progressive airflow limitation along with chronic airway inflammation and systemic effects leading to impaired lung function, shortness of breath, tiredness, and poor QoL (3, 4). Although there are drugs that can help with your breathing problems (long acting bronchodilators and inhaled steroids), these don't work for all the other problems you might have, such as weak muscles, trouble exercising, and mental health issues (5). Therefore, it is considered that comprehensive pulmonary rehabilitation (PR) programs including exercise training, health education, and behavior modification are usually regarded as standard of care to improve functional capacity and quality of life (QoL) in COPD patients (6, 7).

In the last 20 years, there has been growing interest in complementary and alternative exercises which place emphasis on breathing control, gentle movements, and mind-body unification. And these activities are called traditional Chinese mind-body exercises altogether (TCMBEs), encompass Tai Chi, Baduanjin, Liuzijue, Wuqinxi, and Yijinjing, which are ancient regimens designed to synchronize respiration, posture, and mental focus (8). Unlike higher-intensity aerobic or resistance training, TCMBEs involve low to moderate movement that is performed along with breath work, making it ideal for elderly people or individuals with COPD or other physical limitations (9). Mind-body exercises could possibly improve lung function by making the breathing muscles stronger with diaphragmatic breathing, letting your chest move more freely, and helping air switch better (10, 11). They act on the autonomic nervous system to increase parasympathetic activity and reduce sympathetic overactivity, which lowers the respiratory rate and improves dyspnea (12). Also, data shows that they can decrease the whole body inflammation and oxidative damage, which are important parts of COPD, so they give them good things at the molecular level that go past just the lungs (13).

In the first few years of 2,000, the clinical studies conducted showed that mind body therapies did provide some real benefits for those with COPD. Randomized trials have shown that tai chi can improve 6MWD and reduce symptom burden compared to usual care (9). Baduanjin programs were seen to improve lung capacity, exercise stamina, and QoL when done along with regular PR (14, 15). Liuzijue is famous for its respiratory phonation training that can improve FEV1 and functional capacity (16). And meta-analyses agree here as well: In systematic reviews we see that Tai Chi and Qigong types of exercise greatly improve exercise tolerance and health-related quality of life (HRQoL) (14, 17). TCMBEs might bring about significant psychological/social outcomes besides physical ones. COPD is often accompanied by anxiety, depression, and social withdrawal. The thoughtful and careful parts of these actions made it easier for people to control how they felt and have confidence in themselves, so they were better at following their long-term recovery plans(18). Mindfulness based movement raises prefrontal cortex activity and reduces the feeling of being stressed so it lowers behavior risk factors for bad results (19).

Despite much research there are still many gaps. The reported effects vary greatly because of different types of exercises, frequencies of training, amounts of supervision, and participant features, so it limits how generally we can apply them clinically. It is unclear which TCMBE modality works best compared to others as most past studies or meta-analyses focused on one activity such as tai chi or baduanjin (15, 17). Although several studies have been conducted, most originate from East Asian countries, which limits the generalisability and wider applicability of the findings. Moreover, many investigations have overlooked objective indicators such as inflammatory cytokine levels and autonomic function markers, as well as important functional and psychological outcomes. This omission makes it difficult to explain the mechanisms underlying the reported benefits. To address these limitations, a comprehensive synthesis of high-quality randomized controlled trials is required to evaluate the overall and comparative effects of TCMBE on lung function, exercise tolerance, and quality of life in patients with COPD. The aim of this meta-analysis is to provide stronger evidence supporting the integration of mind-body practices into evidence-based pulmonary rehabilitation.

## Methods

This systematic review and meta-analysis was carried out according to PRISMA 2020 guidelines as well as the Cochrane handbook for systematic reviews of interventions (20). The study protocol was registered prospectively in the International Prospective Register of Systematic Reviews (PROSPERO) with registration number CRD420251176312.

### Search strategy

A wide-ranging electronic search was done using PubMed, Embase, Web of Science, Cochrane Library, and Scopus between the start of each database up until 15th October 2025. Search used MeSH and also free text terms relating to diseases and exercise interventions: (“Tai Chi” OR “Taiji” OR “Baduanjin” OR “Liuzijue” OR “Yijinjing” OR “Wuqinxi” OR “Qigong” OR “Health Qigong” OR “Mind–Body Exercise”) AND (“Chronic Obstructive Pulmonary Disease” OR “COPD” OR “Chronic Bronchitis” OR “Emphysema”). And also I looked at the references of already published meta-analysis as well as clinical guidelines for relevant studies. Two reviewers did separate database searches and gathered all the found papers using EndNote X9 to get rid of repeats.

### Eligibility criteria

The inclusion and exclusion criteria were established based on the PICOS framework (Population, Intervention, Comparison, Outcomes, and Study design). Eligible participants were those who were 40 years old or older and had been diagnosed with stable COPD according to the Global Initiative for Chronic Obstructive Lung Disease (GOLD) criteria (post-bronchodilator FEV_1_/forced vital capacity (FVC) < 0.70). The participants had to be clinically stable at least 6 weeks prior to the start of the intervention and able to exercise safely. The intervention comprised TCMBEs, which included Tai Chi (Taijiquan), Baduanjin (Eight-Section Brocade), Liuzijue (Six Healing Sounds), Wuqinxi (Five-Animal Frolics), and Yijinjing (Muscle–Tendon Change Classic). Intervention was done alone or together with medicine treatment or traditional care. Qualified programs last for at least 4 weeks and have sessions that take place 2 or more times per week, lasting for no less than 30 min. Control group got usual care, standard medical management, or other non-mind-body interventions (e.g., walking training, breathing exercises, traditional PR). Inclusion required the reporting of at least one of the following outcome measures: Pulmonary function: FEV_1_, FVC, FEV_1_/FVC ratio, and FEV_1_ as a percentage of the anticipated value. Exercise tolerance: Six-min Walk Distance (6MWD) or Incremental Shuttle Walk Test (ISWT). Patient-reported outcomes: COPD Assessment Test (CAT), St. George's Respiratory Questionnaire (SGRQ), Chronic Respiratory Disease Questionnaire (CRQ), or Modified Medical Research Council (mMRC) Dyspnea Scale. The exclusion criteria were: 1) Non-RCT studies such as observation, quasi-experiment, cross-section design etc., 2) Patients who are in the acute exacerbation phase or have severe comorbidities that affect mobility, 3) Mixed or unspecified exercise protocol 4) Interventions lasting less than 8 weeks 5) Insufficient or duplicate quantitative data 6) High methodological risk of bias.

### Data extraction

And two separate evaluators extracted relevant information using a standard data form. Information about studies was collected (authors, publication year, country), participant attributes (sample size, age, disease severity, and gender distribution), intervention specifics (exercise type, frequency, session length, and total duration), control conditions, and primary outcomes at baseline and post-intervention. In the absence of essential statistical data (e.g., standard deviations or sample sizes), estimates came from other reported stats or we asked the study authors for their thoughts by email. All obtained information was confirmed by a third person to guarantee accuracy.

### Risk of bias assessment

The methodological quality of the included studies was evaluated separately by two reviewers using the Cochrane Risk of Bias 2.0 (RoB 2.0) tool (21). Examined domains comprise (1) bias due to the randomization technique, (2) deviation from intended interventions, (3) missing outcomes, (4) methods for assessing outcomes, and (5) selective results presentation. Any discrepancies were rectified through consensus or contact with a senior reviewer.

### Statistical analysis

All statistical analyses were conducted utilizing R software (version 4.3.2, R Foundation for Statistical Computing, Vienna, Austria) along with the ‘meta', ‘metafor', and ‘dmetar' packages. For pooled results on continuous outcomes, MDs or SMDs along with 95% CIs were used. I^2^ statistic and Cochran's Q test were performed to check the degree of heterogeneity among studies. I^2^ values of 25%, 50%, and 75% indicate minimal, moderate, and high heterogeneity, respectively. A random-effects model (DerSimonian–Laird approach) was utilized in the presence of significant heterogeneity (I^2^ > 50%); A fixed effects model was used Sensitivity analyses were done by removing each study one at a time. Subgroup analysis was pre-determined by type of exercise (e.g., Tai Chi, Baduanjin, Liuzijue). Publication bias was evaluated through the symmetry of a funnel plot and Egger's regression test; a *p*-value < 0.05 indicated the presence of bias. Effect sizes were plotted using forest plots and funnel plots were generated in R to show effect size distribution and variation. All statistical tests were two-tailed and results were considered statistically significant when *p* < 0.05.

## Results

### Study selection

**Figure 1:**
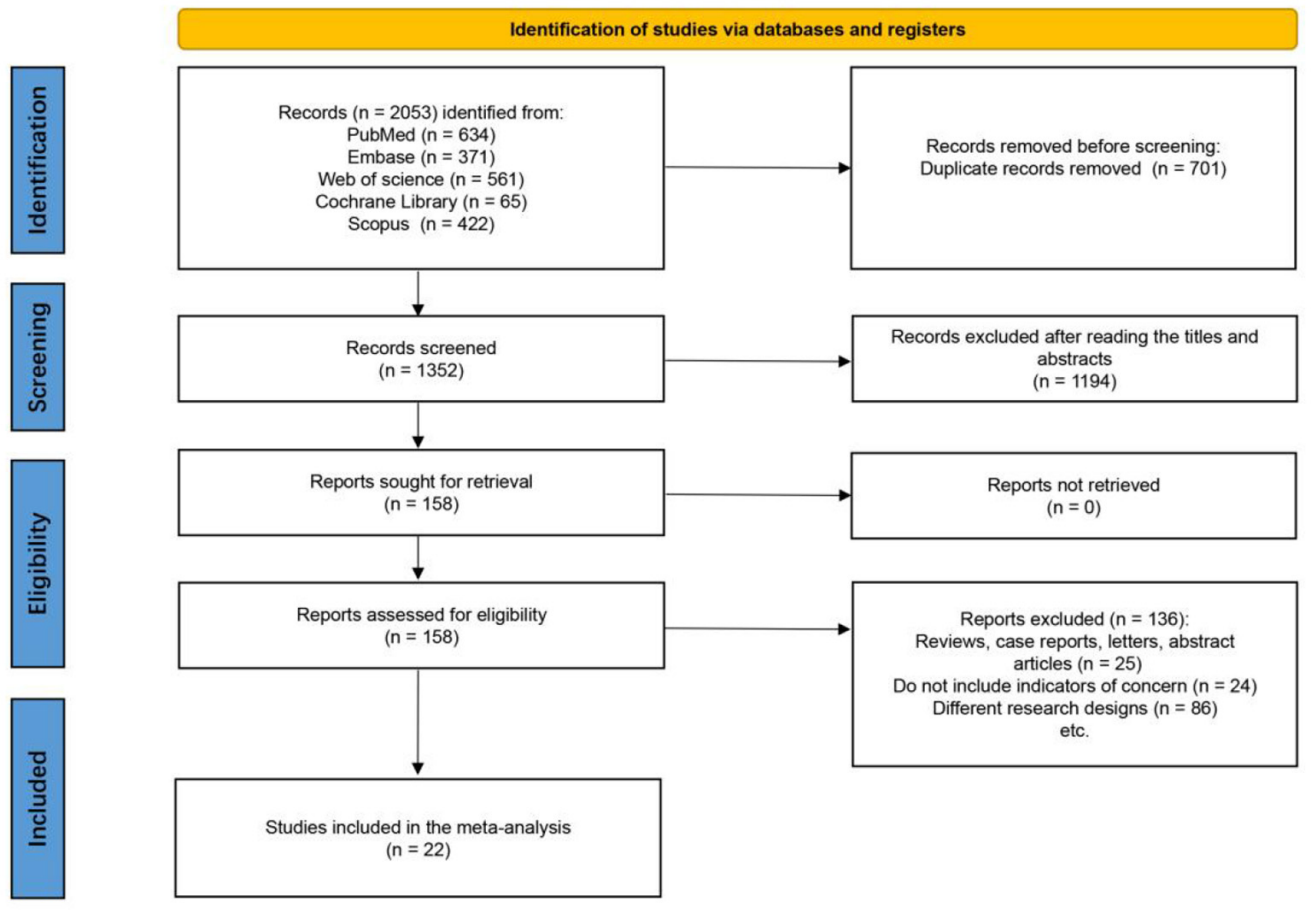
PRISMA flow diagram of study selection for the meta-analysis.

The preliminary electronic search across five international databases—PubMed (*n* = 634), Embase (*n* = 371), Web of Science (*n* = 561), Cochrane Library (*n* = 65), and Scopus (*n* = 422)—produced a cumulative total of 2,053 records were produced. After removing 701 duplicates, there were 1,352 studies left that had titles and abstracts screened. Out of which 1,194 papers were excluded as they did not fulfill the inclusion criteria or were not related to TCMBEs in COPD patients. After the first screening, 158 reports were screened out for the whole text review. No report was ruled out because no full text was available. After the thorough assessment of the texts, 136 articles were ruled out due to different reasons: 25 were reviews, case reports, letters, or conference abstracts; 24 did not have important results such as lung function or ability to exercise; and 86 were not randomized controlled trials. All together, 22 RCTs (22–43) met the qualification standards have been included in the final quantitative Meta-analysis. Identification, screening, eligibility assessment, and lastly incorporation into the overall process is shown in Figure 1 according to the PRISMA 2020 reporting method.

### Study characteristics

This meta-analysis comprised 22 RCTs (22–43) with 1,871 patients with COPD. All papers were published between 2010 and 2025, showing that there was continued study on integrating TCMBEs into PR. Most of them were done in China (*n* = 17), supplemented by studies from the United States (*n* = 4) and Thailand (*n* = 1). All included trials were parallel group RCTs comparing TCMBEs used together with regular medical treatment or usual care to controls getting standard care, education, or typical rehabilitation. Different studies had different sample size, it was between 10 and 318 people, the number of people in the experiment group was between 5 and 158 and the number of people in the control group was between 5 and 160. The mean age for all participants ranged from 59.7 to 72.2, most were male, this is typical of COPD groups. The interventions encompassed five primary TCMBE modalities: (1) Liuzijue Six Healing Sounds; *n* = 7, (2) Tai Chi Taijiquan; *n* = 9, (3) Baduanjin Eight-Section Brocade; *n* = 3, (4) Yijinjing Muscle–Tendon Change Classic; *n* = 1), and (5) blended or combined TCMBE protocols (*n* = 2).

Training happened roughly 2–5 times each week, and session length was anywhere from 15–60 min depending on what kind of exercise it was and how the study was designed. Most lasted 8–24 weeks, some were over 6 months, allowing enough time for physiologic adaption and rehabilitation benefits. Control condition(s) showed slight variations between each trial; most often it was just usual pharmaceuticall treatment, health eduction, or usual care without any exercize program. Some trials used active control such as mind-body breathing, walking, or traditional PR as a comparison group for exercise-specific benefits. The outcomes measures from the included RCTs were all different but also complementary. All of them did lung function tests, mostly FEV_1_, FVC, FEV_1_/FVC or FEV_1_%pred. Exercise capacity was mainly evaluated by 6MWD test. QoL and symptom-related measures, such as the SGRQ, Chronic Respiratory Questionnaire (CRQ), CAT, and mMRC Dyspnea Scale, And there's also a greater emphasis on patient-centred outcomes. Lots of experiments did this at once, so they could look at changes in the body as well as people's feelings. But there was still some difference between trials in terms of type of exercise, how closely they watched for how hard someone was working, and how long they followed up. In Table 1, a summary of all the included studies is given with information about where the studies come from, who participated, what the studies did, and what they found out.

**Table 1:** Characteristics of included studies on traditional Chinese mind-body exercises in COPD rehabilitation.

**Study**	**Study Design**	**Country**	**Intervention**		**Frequency**	**Duration**	**Sample size** ***(n)***		**Age (years)**		**Gender (M/F)**		**Outcome measures**
			**EG**	**CG**			**EG**	**CG**	**EG**	**CG**	**EG**	**CG**	
Yang 2025	RCT	China	Baduanjin plus standard medical treatment	Standard medical treatment	15 min/time, 3 times/week	12 weeks	20	25	69.20 ± 3.97	68.56 ± 4.83	7/13	15/10	FEV1, FVC, FEV1/FVC (%), CAT
Chen 2025	RCT	China	Baduanjin	Routine healthcare	30 min/time, 2 times/day, 5 days/week	12 weeks	55	53	66.7± 6.7	66.7± 8.1	45/10	37/16	FEV1%, 6MWD, FEV1/FVC (%), CAT, SGRQ
Chen 2024	RCT	China	Baduanjin plus usual care	Usual care	30 min/time, 2 times/day, 5 days/week	6 months	158	160	61.52 ± 10.31	61.97 ± 10.91	89/69	88/72	6MWD, CAT, mMRC
Zhang 2016	RCT	China	Yijinjing	Usual care	60 min/time, 3 times/week	6 months	42	45	64.77 ± 11.07	62.35 ± 9.27	33/9	35/10	FEV1, FEV1%, FEV1/FVC (%), 6MWD, CAT
Zheng 2022	RCT	China	Liuzijue plus usual care	Usual care	30 min/time, 2 times/day, 7 days/week	4 weeks	50	50	68.21 ± 2.65	68.26 ± 2.54	39/11	40/10	FEV1, FVC, FEV1/FVC (%)
Wu 2020	RCT	China	Liuzijue plus usual care	Usual care	60 min/time, 6 times/week	6 months	16	17	67 ± 8	66 ± 9	14/2	14/3	FEV1, FEV1%, FEV1/FVC (%), 6MWD, SGRQ
Liao 2021	RCT	China	Liuzijue plus usual care	Usual care	30 min/time, 2 times/day, 7 days/week	3 months	34	36	61.83 ± 6.63	61.21 ± 7.38	28/6	27/9	FEV1%, FEV1/FVC (%), 6MWD, SGRQ, mMRC
Wu 2018	RCT	China	Liuzijue plus standard medical treatment	Standard medical treatment	60 min/time, 2 times/week	2 months	32	33	65 ± 11	65 ± 8	9/5	12/4	FEV1%, FEV1/FVC (%)
Li 2021	RCT	China	Liuzijue plus usual care	Usual care	60 min/time, 6 times/week	6 months	17	19	66 ± 9	66 ± 9	14/3	14/5	FEV1%, FEV1/FVC (%), 6MWD, SGRQ
Liu 2021	RCT	China	Liuzijue plus usual care	Usual care	60 min/time, 2 times/week	12 weeks	14	16	65 ± 11	66 ± 8	9/5	12/4	6MWD, SGRQ
Xiao 2015	RCT	China	Liuzijue plus standard medical treatment	Standard medical treatment	45 min/time, 4 times/week	6 months	63	63	72.2 ± 1.7	70.9 ± 1.4	58/5	59/4	6MWD
Yeh 2020	RCT	USA	Tai-chi	Education	60 min/time, 2 times/week	12 weeks	61	31	68.6 ± 9.2	68.1 ± 6.7	43/18	18/13	6MWD, CAT
Kantatong 2020	RCT	Thailand	Tai-chi	Usual care	3 times/week	12 weeks	25	25	69.68 ± 7.67	67.48 ± 10.17	15/10	19/6	FEV1, FVC, 6MWD, SGRQ, mMRC
Chan 2013	RCT	China	Tai-chi	Usual care	60 min/time, 2 times/week	3 months	70	67	71.7 ± 8.2	73.6 ± 7.4	69/1	58/9	SGRQ
Kraemer 2021	RCT	USA	Tai-chi	Mind-body breathing	60 min/time, 2 times/week	12 weeks	61	31	68.6 ± 9.2	67.5 ± 7.7	43/18	18/13	6MWD, CAT
Moy 2021	RCT	USA	Tai-chi	Usual care	60 min/time, 2 times/week	12 weeks	36	37	69.6 ± 7.5	70.5 ± 9.2	17/19	27/10	6MWD, CAT
Polkey 2018	RCT	China	Tai-chi	Pulmonary Rehabilitation	60 min/time, 5 times/week	12 weeks	60	60	40–80	40–80	-	-	FEV1, FVC, 6MWD, SGRQ, mMRC
Liu 2023	RCT	China	Tai-chi	Usual care	30 min/time, 3 times/week	2 months	26	26	66.27 ± 6.58	60.77 ± 7.48	19/7	18/8	FEV1, FEV1%, FVC, 6MWD, SGRQ, mMRC
Yeh 2010	RCT	USA	Tai-chi plus usual care	Usual care	60 min/time, 2 times/week	12 weeks	5	5	65 ± 6	66 ± 6	3/2	3/2	6MWD, CAT, FEV1/FVC (%)
Chan 2011	RCT	China	Tai-chi	Usual care	60 min/time, 2 times/week	3 months	70	67	71.7 ± 8.2	73.6 ± 7.4	69/1	58/9	FEV1, FVC, 6MWD
Niu 2014	RCT	China	Tai-chi	Standard medical treatment	30 min/time, 7 times/week	6 months	20	20	59.7 ± 2.76	61.3 ± 2.89	19/1	18/2	FEV1, FEV1%, 6MWD
Wang 2018	RCT	China	Tai-chi	Usual care	60 min/time, 3 times/week	3 months	26	24	67.83 ± 5.32	67.86 ± 5.98	23/3	22/2	FEV1, FEV1%, FEV1/FVC (%), 6MWD, CAT

EG, experimental group; CG, control group; FEV1, forced expiratory volume in 1 s; FVC, forced vital capacity; FEV1/FVC, forced expiratory volume in 1 s/forced vital capacity; FEVI%, the percent of forced expiratory volume in one s in prediction; CAT, the COPD Assessment Test questionnaires; 6MWD, 6-min walk distance; SGRQ, St. George's respiratory questionnaire; mMRC, modified medical research council dyspnea scales.

### Risk of bias assessment

**Figure 2:**
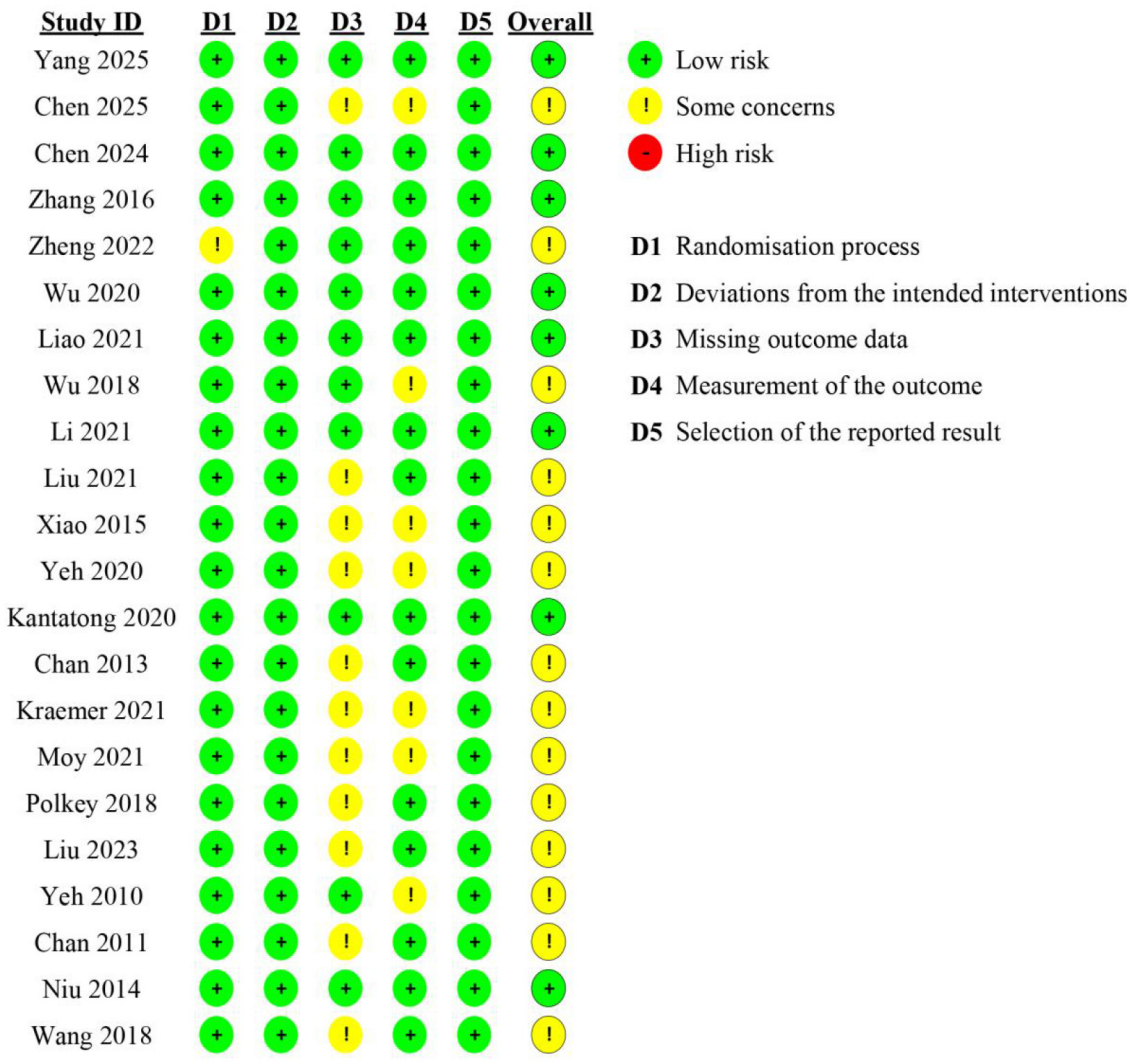
Risk of bias assessment for included studies (Cochrane RoB 2.0 tool).

The methodological quality of the included RCTs was evaluated by using the Cochrane RoB 2.0 tool, and the summary is shown in Figure 2. In terms of the 22 studies analyzed, 11 (50%) had a low overall risk of bias, whereas 11 (50%) had some concern. All studies were considered to have a low overall risk of bias. First up is the first domain- bias due to the randomization process (D1) - where most of the studies (95%) showed enough random sequence generation and allocation concealment, which means their methods were pretty good. Only one publication (43). There was some trouble due to not enough information about how things were given out. Second domain–Bias due to departures from the intended intervention (D2)- all trials were considered at low risk. Most trials mentioned adherence tracking and instructors watching over people doing the workouts, which means most people probably did the workouts the way they were told to do them. The third domain is bias due to missing outcomes data (D3). About half of the studies had moderate concerns regarding this type of bias (13 out of 22). The researches did not explain attrition sufficiently or gave no thorough explanation as to why participants left. But still, dropout was below 15% in most of the studies, so there's less chance for big outcome bias. As far as the fourth domain-bias in outcome measurement (D4)-concerns, all but 2 studies were considered at low risk. Most trials had objective measures of lung function (FEV_1_, FVC) and validated tools such as CAT, SGRQ which were evaluated by blind assessors in clinical/rehab settings. In D5 Bias in Selection of Reported Result, 82% of the studies had prespecified outcomes that were stated and reported as such by the majority of the studies. But there are some older trials that do not specify the outcome in advance (23, 31) lacked registration details or complete statistical reporting, leading to a classification of “some concerns.” The methodological rigor of the papers which have been included was found to be moderate to high; therefore, the results which have been combined here in this meta-analysis are from papers of good enough quality. Figure 2 gives the visual depiction of the domain specific risk of bias assessment of all the studies included.

### GRADE assessment for the main outcomes

We assessed the certainty of evidence for the main outcomes using GRADE (Table 2). Overall, traditional Chinese mind–body exercises were associated with favorable effects on lung function, exercise capacity, and health-related quality of life compared with control interventions. The certainty of evidence was mostly moderate, with high certainty for the FEV1/FVC outcome. Across outcomes, downgrading was mainly due to inconsistency (between-study heterogeneity), while risk of bias, indirectness, and imprecision were generally judged as not serious. For lung function, evidence from 10 randomized trials (intervention: 405; control: 406) showed that FEV1 improved by MD 0.20 (95% CI 0.09–0.30), with moderate certainty due to serious inconsistency. FVC, based on 6 trials (251 vs. 253), also improved (MD 0.22, 95% CI 0.06 to 0.38; moderate certainty), again downgraded for inconsistency. The FEV1/FVC ratio, derived from 10 trials (279 vs. 290), increased by MD 3.42 (95% CI 2.54–4.29) with high certainty. FEV1% predicted improved in nine trials (300 vs. 303) by MD 4.98 (95% CI 2.54–7.43), with moderate certainty, downgraded for inconsistency. Regarding functional capacity, 18 trials (839 vs. 782) reported 6MWD and showed an increase of MD 42.05 m (95% CI 29.06–55.05), with moderate certainty after downgrading for inconsistency. For health status and symptoms, SGRQ (nine trials; 317 vs. 319) decreased (improved) by MD −13.76 (95% CI −20.83– −6.68) with moderate certainty, downgraded for inconsistency. CAT (9 trials; 464 vs. 411) also improved, with a reduction of MD −2.62 (95% CI −4.68–−0.50), supported by moderate certainty. Dyspnea assessed by mMRC (four trials; 277 vs. 281) decreased by MD −0.50 (95% CI −0.84–−0.15), with moderate certainty, again downgraded due to inconsistency.

**Table 2:** GRADE assessment for the main outcomes.

**No. of studies**	**Certainty assessment**						**No. of patients**		**Effect**		**Certainty**
	**Study design**	**Risk of bias**	**Inconsistency**	**Indirectness**	**Imprecision**	**Other considerations**	**Combination**	**Control**	**Relative (95% CI)**	**Absolute (95% CI)**	
**FEV1**											
10	Randomized trials	Not serious	Serious	Not serious	Not serious	None	405	406	–	MD 0.20 higher (0.09 higher−030 higher)	⊕⊕⊕○ Moderare
**FVC**											
6	Randomized trials	Not serious	Serious	Not serious	Not serious	None	251	253	–	MD 0.22 higher (0.06 higher−0.38 higher)	⊕⊕⊕○ Moderare
**FEV1/FVC**											
10	Randomized trials	Not serious	Not serious	Not serious	Not serious	None	279	290	–	MD 3.42 higher (2.54 higher−4.29 higher)	⊕⊕⊕⊕ High
**FEV1%**											
9	Randomized trials	Not serious	Serious	Not serious	Not serious	None	300	303	–	MD 4.98 higher (2.54 higher−7.43 higher)	⊕⊕⊕○ Moderare
**6MWD**											
18	Randomized trials	Not serious	Serious	Not serious	Not serious	None	839	782	–	MD 42.05 higher (29.06 higher−55.05 higher)	⊕⊕⊕○ Moderare
**SGRQ**											
9	Randomized trials	Not serious	Serious	Not serious	Not serious	None	317	319	–	MD 13.76 lower (20.83 lower−6.68 lower)	⊕⊕⊕○ Moderare
**CAT**											
9	Randomized trials	Not serious	Serious	Not serious	Not serious	None	464	411	–	MD 2.62 lower (4.68 lower−0.5 lower)	⊕⊕⊕○ Moderare
**mMRC**											
4	Randomized trials	Not serious	Serious	Not serious	Not serious	None	277	281	–	MD 0.50 lower (0.84 lower−0.15 lower)	⊕⊕⊕○ Moderare

FEV1, forced expiratory volume in 1 s; FVC, forced vital capacity; FEV1/FVC, forced expiratory volume in 1 s/forced vital capacity; FEVI%, the percent of forced expiratory volume in one second in prediction; CAT, the COPD Assessment Test questionnaires; 6MWD, 6-min walk distance; SGRQ, St. George's Respiratory Questionnaire; mMRC, Modified Medical Research Council Dyspnea Scales.

### Effects of traditional Chinese mind–body exercises on FEV_1_

**Figure 3:**
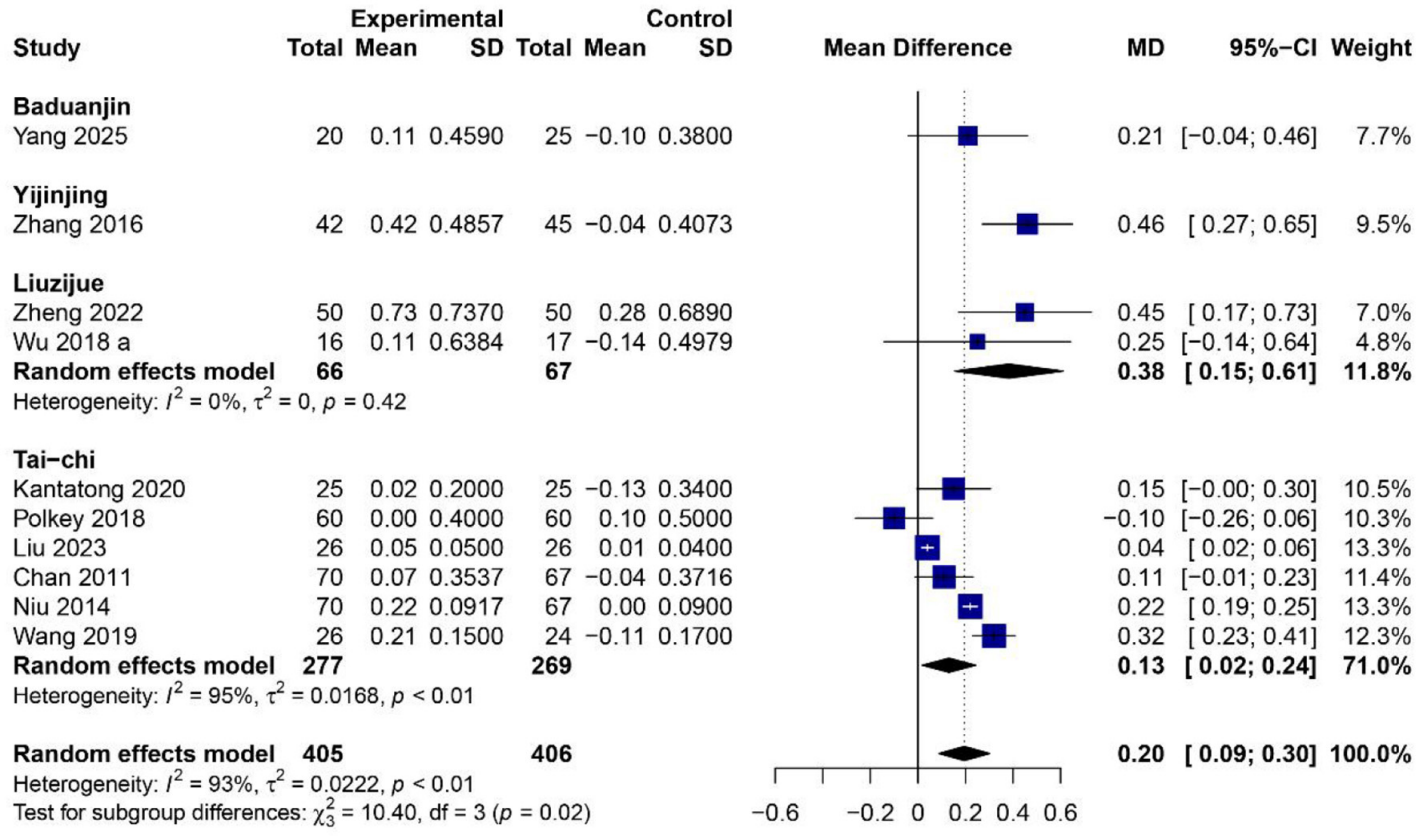
Forest plot of subgroup meta-analysis for the effect of TCMBEs on FEV_1_ in patients with COPD.

Figure 3 shows the combined effect of 4 exercise modes (Baduanjin, Yijinjing, Liuzijue, Tai Chi) on FEV1 improvement, obtained from information in 10 (RCTs (23, 26, 30, 33–36, 39, 42, 43) including 811 participants (405 in the intervention group and 406 in the control group). In summary, TCMBEs markedly improved FEV_1_ relative to control interventions, with a total mean difference (MD) of 0.20 L (95% CI: 0.09–0.30, *p* < 0.01) according to a random-effects model. The included studies exhibited considerable heterogeneity (*I*^2^ = 93%, *p* < 0.01), indicating variety in the type, intensity, and duration of exercise. Subgroup analysis indicated significant disparities among exercise modalities (χ^2^ = 10.40, *df* = 3, *p* = 0.02). Liuzijue had the greatest significant impact (*MD* = 0.38 L, 95% CI: 0.15–0.61, *p* < 0.01; *I*^2^ = 0%), followed by Yijinjing (*MD* = 0.46 L, 95% CI: 0.27–0.65) and Baduanjin (*MD* = 0.21 L, 95% CI: −0.04–0.46), Both improved lung function compared to controls Tai Chi had a small but significant improvement (*MD* = 0.13 L, 95% CI: 0.02–0.24, *p* < 0.05; *I*^2^ = 95%), seems that it could be due to differences in the amount of time spent exercising and different methods. The collected evidence shows that TCMBEs can bring a certain degree of improvement to lung function for people with COPD, especially those that pay attention to breath control and coordinated body movements such as Liuzijue and Yijinjing.

### Effects of traditional Chinese mind–body exercises on FVC

**Figure 4:**
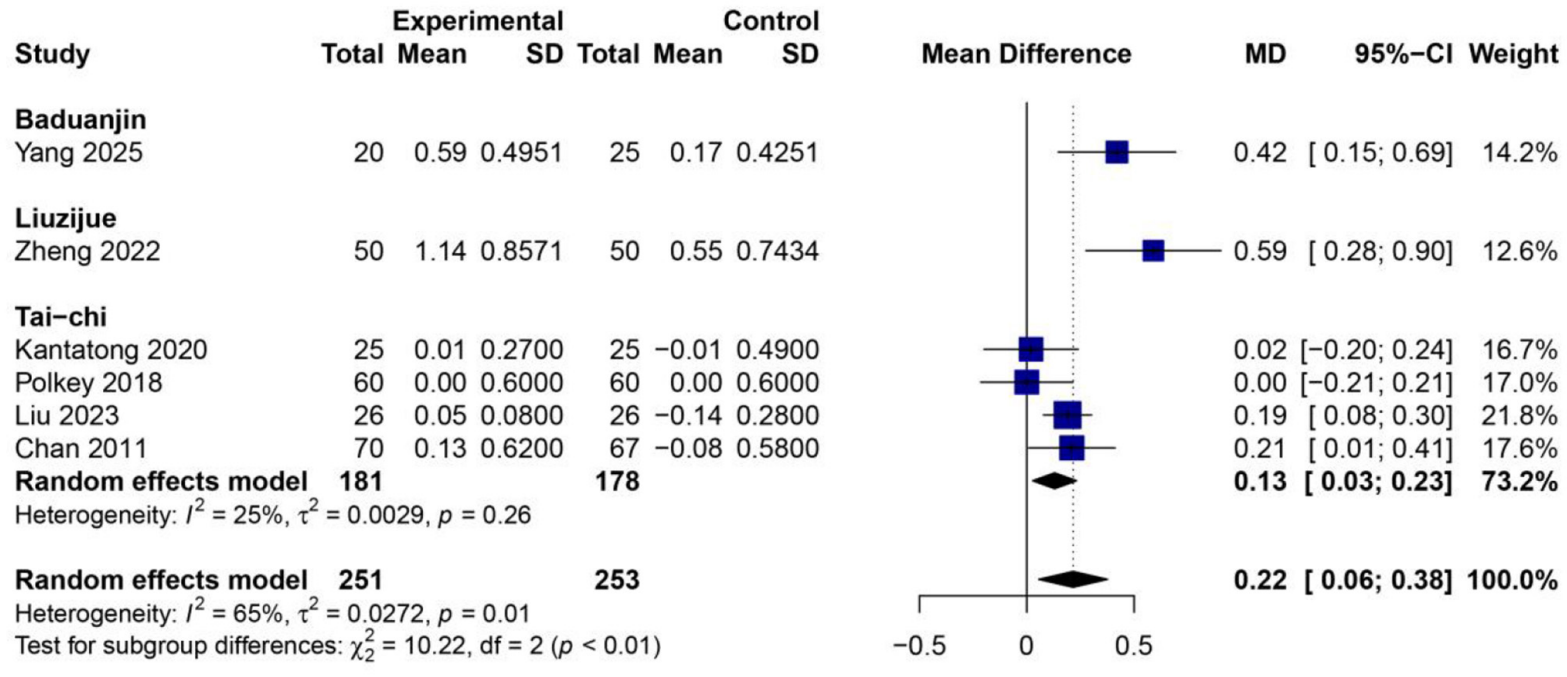
Forest plot of subgroup meta-analysis for the effect of TCMBEs on FVC in patients with COPD.

The pooled analysis of six RCTs (23, 26, 30, 34, 39, 43) (*n* = 504) TCMBEs resulted in greater improvements in FVC than standard care or routine rehabilitation MD 0.22 L (95% CI: 0.06–0.38, *p* < 0.01) under a random-effects model. Moderate heterogeneity was detected (*I*^2^ = 65%, τ^2^ = 0.0272, *p* = 0.01). Subgroup analysis showed there were differences between different kinds of exercises (χ^2^ = 10.22, *df* = 2, *p* < 0.01). Liuzijue demonstrated the most substantial enhancement in FVC (*MD* = 0.59 L, 95% CI: 0.28–0.90, *p* < 0.01), closely succeeded by Baduanjin (*MD* = 0.42 L, 95% CI: 0.15–0.69, *p* < 0.01), signifying notable advantages for lung expansion and respiratory capacity. Tai Chi exhibited a modest yet significant pooled impact (*MD* = 0.13 L, 95% CI: 0.03–0.23, *p* < 0.05; *I*^2^ = 25%; Figure 4), Moderate-intensity exercise can still bring about measurable improvements even if there are differences in the amount of time spent on the program.

### Effects of traditional Chinese mind–body exercises on FEV_1_/FVC ratio

**Figure 5:**
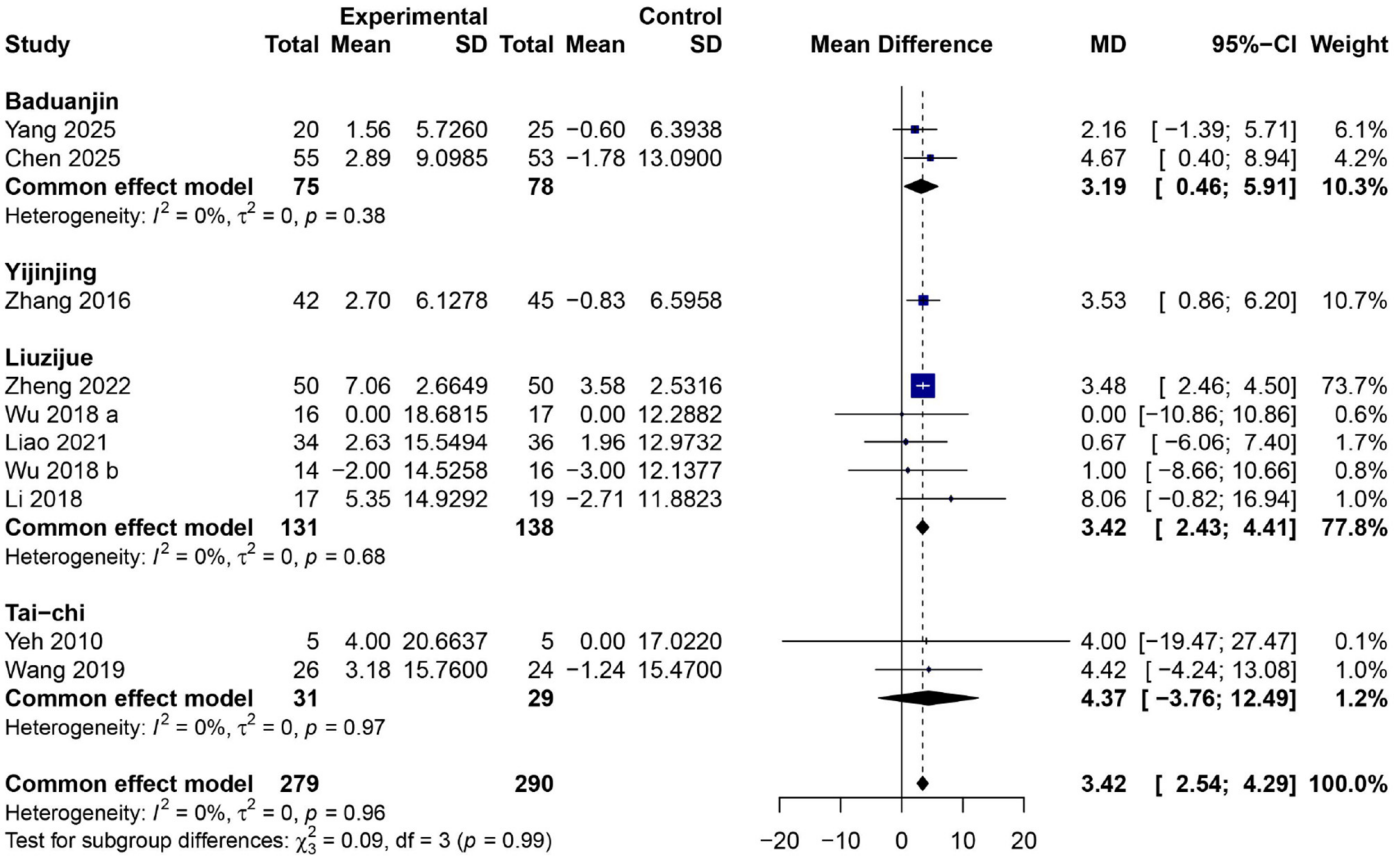
Forest plot of subgroup meta–analysis for the effect of TCMBEs on the ratio of FEV_1_ to FVC in patients with COPD.

Figure 5 presents the aggregate findings from 10 RCTs, which show that taking part in TCMBEs led to a noticeably better FEV_1_/FVC ratio compared to normal conditions (24, 28, 29, 35–37, 39, 41–43) encompassing 571 people (experimental = 281, control = 290). The whole combined MD was 3.42 (95% CI: 2.54–4.29, *p* < 0.01), signifying a moderate yet significant enhancement in airflow efficiency subsequent to TCMBE exercise. The heterogeneity among trials was minimal (*I*^2^ = 0%, τ^2^ = 0, *p* = 0.95), indicating remarkably consistent outcomes. Subgroup analyses revealed similar enhancements across several exercise modalities, with Liuzijue yielding a pooled MD of 3.42 (95% CI: 2.43–4.41), Baduanjin 3.19 (95% CI: 0.46–5.91), Yijinjing 3.53 (95% CI: 0.86–6.20), and Tai Chi 4.37 (95% CI: −3.76–12.49). The χ^2^ test for subgroup differences (χ^2^ = 0.09, *df* = 3, *p* = 0.99) revealed no statistically significant differences among exercise modalities.

### Effects of traditional Chinese mind–body exercises on FEV_1_ % predicted

**Figure 6:**
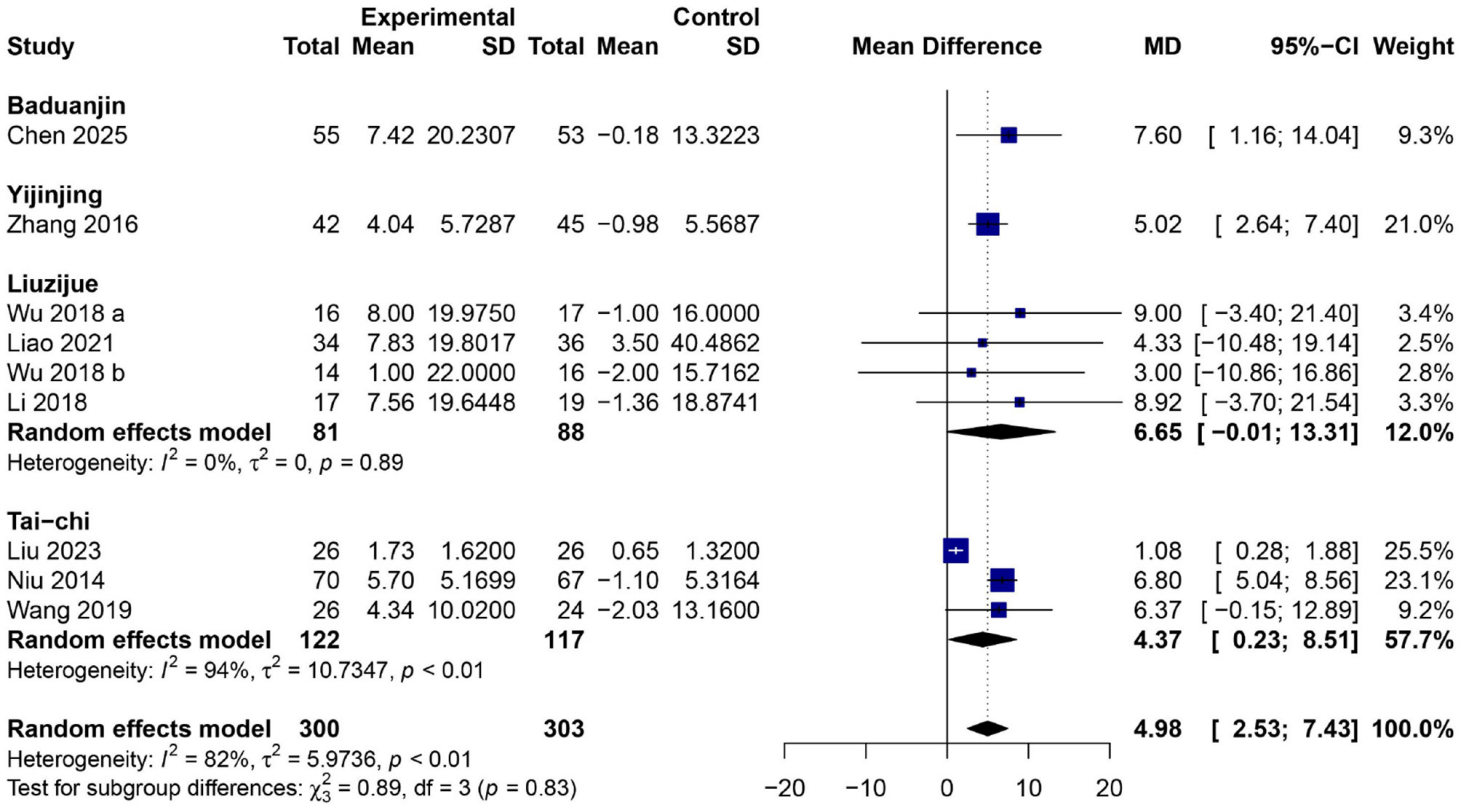
Forest plot of subgroup meta-analysis for the effect of TCMBEs on percent predicted FEV_1_ % in patients with COPD.

The pooled analysis of nine RCTs (24, 28–30, 33, 35–37, 42) (total = 605 participants; 302 in the experimental group and 303 in the control group) showed that taking part in TCMBEs brought about a statistically important improvement in FEV1% predicted. Random-effects model gave an overall MD of 4.98 (95% confidence interval: 2.53–7.42, *p* < 0.01), indicating that there had been considerable improvements in their lung functions compared with the control group. The overall heterogeneity was large (*I*^2^ = 82%, *p* < 0.01), however subgroup analysis revealed no significant inter-group difference (χ^2^ = 0.87, *df* = 3, *p* = 0.83), and it would suggest that there's some kind of uniformly good thing happening for all kinds of exercise. Within the subgroups, Liuzijue exhibited the most significant enhancement (*MD* = 6.57, 95% CI: −0.02–13.16), succeeded by Baduanjin (*MD* = 7.60, 95% CI: 1.16–14.04) and Yijinjing (*MD* = 5.02, 95% CI: 2.64–7.40). Tai Chi, albeit somewhat diminished in intensity, yet produced a notable pooled impact (*MD* = 4.37, 95% CI: 0.23–8.51, *p* < 0.05) (Figure 6).

### Effects of traditional Chinese mind–body exercises on six-min walk distance (6MWD)

**Figure 7:**
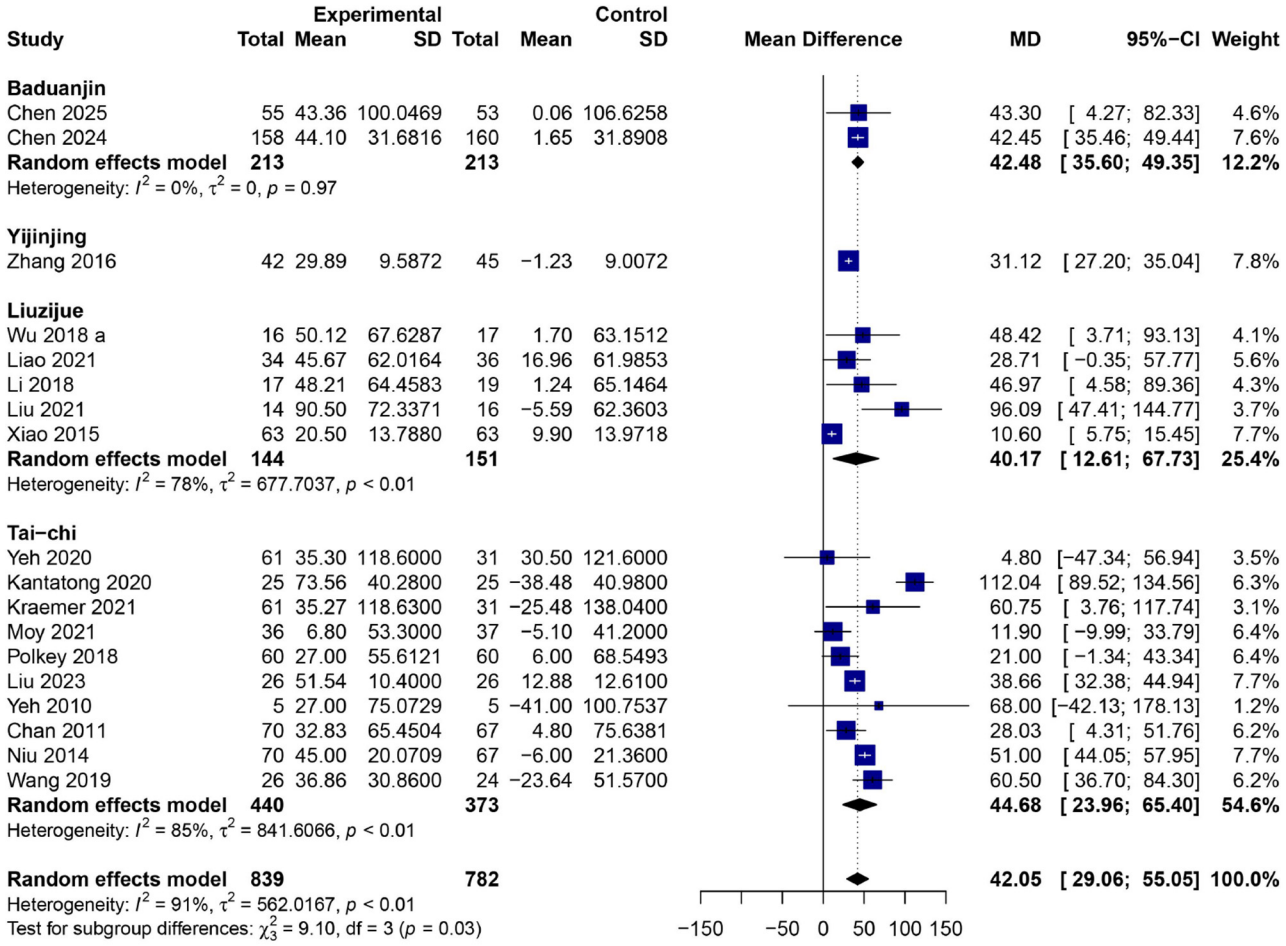
Forest plot of subgroup meta-analysis for the effect of TCMBEs on the six-min walk distance (6MWD) in patients with COPD.

In 18 RCTs (23–36, 38, 40–42) Involving 1,621 participants (839 in the experimental group and 782 in the control group), TCMBEs significantly enhanced the 6MWD relative to control interventions, yielding an overall MD of 42.05 meters [95% confidence interval: 29.06–55.05, *p* < 0.01] (Figure 7) using a random-effects model. Substantial heterogeneity was observed (*I*^2^ = 85%, *p* < 0.01), and subgroup analysis revealed significant differences across various exercise modalities ($\chi_{3}^{2}$ = 9.10, *df* = 3, *p* = 0.03). The most significant enhancement was noted in the Tai Chi subgroup (*MD* = 44.68 m, 95% CI: 23.96–65.40, *p* < 0.01), followed by Liuzijue (*MD* = 40.17 m, 95% CI: 12.61–67.73, *p* < 0.01) and Baduanjin (MD = 42.48 m, 95% CI: 35.60–49.35, *p* < 0.01). The Yijinjing demonstrated a positive result (*MD* = 31.12 m, 95% CI: 27.20–35.04), but derived from a limited number of investigations.

### Effects of traditional Chinese mind–body exercises on St. George's respiratory questionnaire score

**Figure 8:**
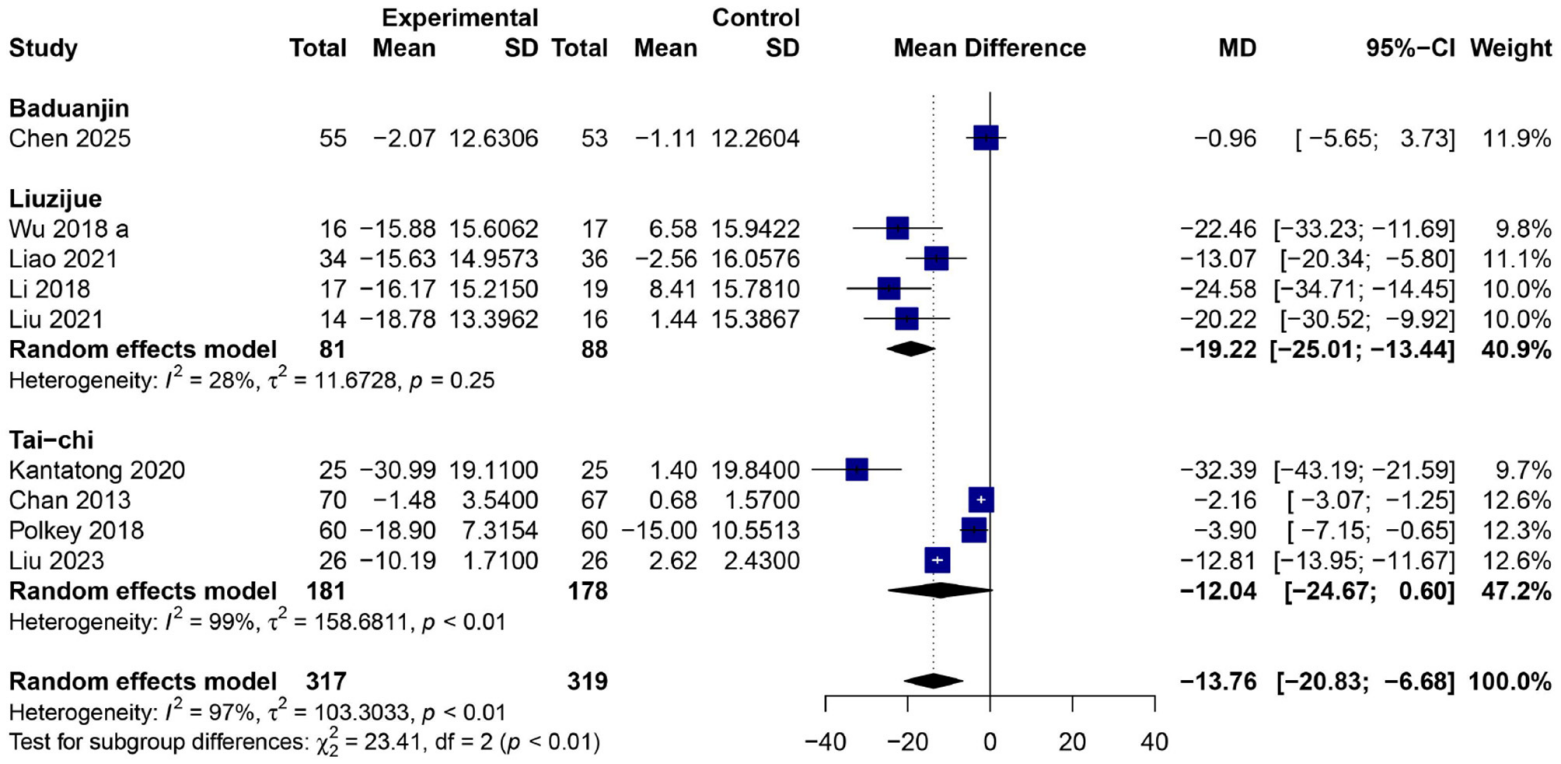
Forest plot of subgroup meta-analysis for the effect of TCMBEs on SGRQ total scores in patients with COPD.

The comprehensive meta-analysis encompassing nine RCTs (22, 24, 26, 28–31, 34, 36) (total = 636 participants; 317 in the experimental group, 319 in the control group) revealed a statistically significant decrease in SGRQ total scores subsequent to TCMBE interventions, with a MD of −13.76 [95% CI: −20.83–−6.68, *p* < 0.01] (Figure 8) utilizing a random-effects model. There was notable heterogeneity (*I*^2^ = 97%, *p* < 0.01) due to differences between the studies' sample populations as well as their methods for intervention. The subgroup analysis showed that Liuzijue had the largest decrease in SGRQ scores [*MD* = −19.22 (95% CI: −25.01–−13.44)], showing significant improvement in how they feel about their ability to breathe and do everyday activities. Tai Chi group showed considerable drop [*MD* = −12.04 (95% CI: −24.67–0.60)], whereas Baduanjin exhibited a negligible yet non-significant reduction [*MD* = −0.96 (95% CI: −5.65–3.73)]. The analysis on subgroups difference is statistically significant (χ^2^ = 23.41, *df* = 2, *p* < 0.01), From the results we can see that TCMBEs such as Liuzijue and Tai Chi have been good methods to help improve the QoL for people with COPD by lowering their respiratory symptoms and helping them with their physical and mental well being.

### Effects of traditional Chinese mind–body exercises on chronic obstructive pulmonary disease assessment test score

**Figure 9:**
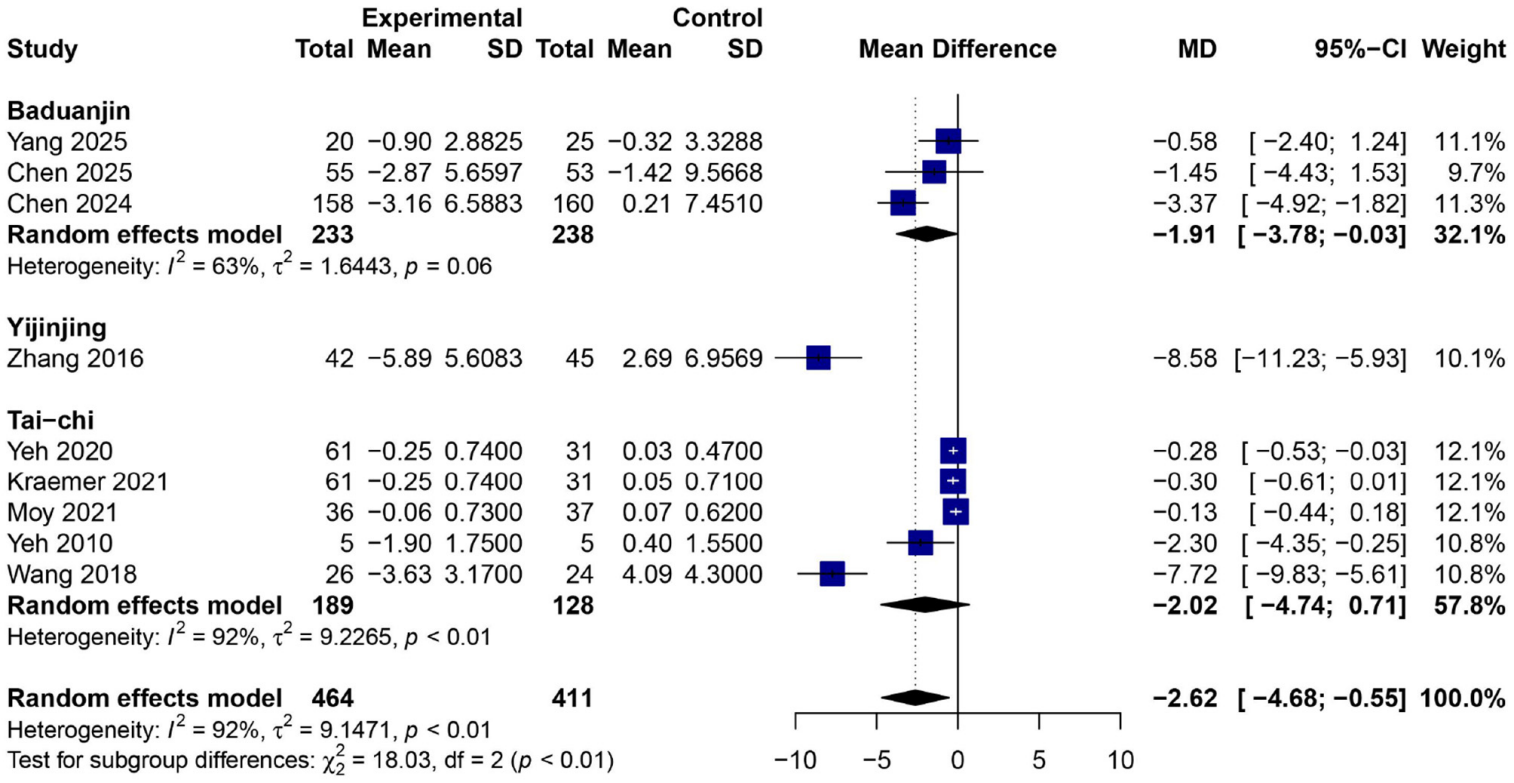
Forest plot of subgroup meta-analysis for the effect of TCMBEs on CAT scores in patients with COPD.

In nine RCTs (24, 25, 27, 32, 35, 39–42) involving a total of 875 participants (464 in the experimental group and 411 in the control group), TCMBEs led to a statistically significant overall reduction in CAT scores relative to controls, with a pooled MD of −2.62 [95% CI: −4.68–−0.55, *p* < 0.01] (Figure 9) using a random-effects model. Significant heterogeneity was observed (*I*^2^ = 92%, *p* < 0.01), indicating variability in training time and participant attributes. The χ^2^ test for subgroup differences was statistically significant (χ^2^ = 18.03, *df* = 2, *p* < 0.01), demonstrating variability among exercise kinds. Subgroup analysis indicated that Yijinjing produced the most significant mean reduction [*MD* = −8.58 (95% CI: −11.23– −5.93)], followed by Tai Chi [*MD* = −2.02 (95% CI: −4.74–0.71)] and Baduanjin [*MD* = −1.91 (95% CI: −3.78–−0.03)].

### Effects of traditional Chinese mind–body exercises on modified medical research council dyspnea score

**Figure 10:**
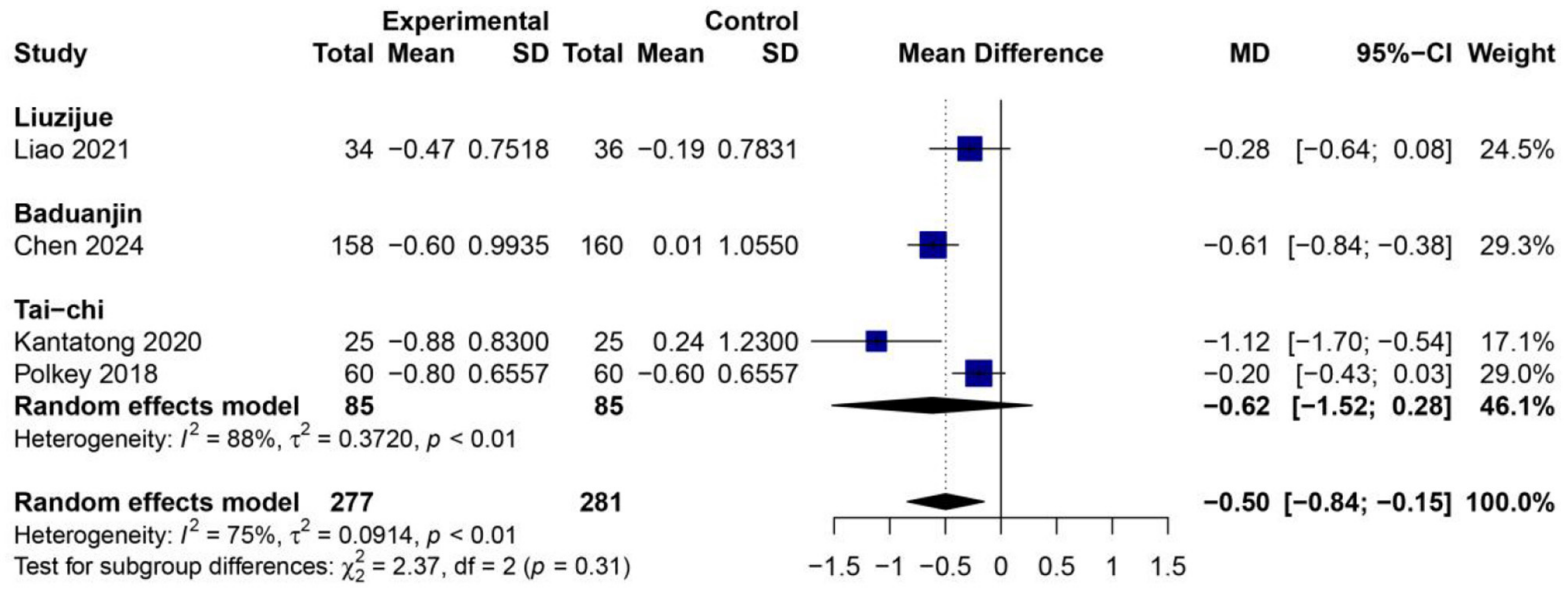
Forest plot of subgroup meta-analysis for the effect of TCMBEs on dyspnea severity measured by the mMRC scale in patients with COPD.

Figure 10 demonstrates that a pooled analysis of four RCTs (25, 26, 29, 34) involving 558 participants (277 in the experimental group and 281 in the control group) indicated that the practice of TCMBEs significantly reduced dyspnea compared to control interventions, resulting in an overall MD of −0.50 [95% CI: −0.84 – −0.15, *p* < 0.01] using a random-effects model. The variability among studies was moderate (*I*^2^ = 75%, *p* < 0.01), however the analysis for subgroup differences revealed no significant disparity between exercise modalities (χ^2^ = 2.37, *df* = 2, *p* = 0.31). Within the subgroups, Tai Chi demonstrated the most significant enhancement [*MD* = −0.62 (95% CI: −1.52–0.28)], followed closely by Baduanjin [*MD* = −0.61 (95% CI: −0.84 – −0.38)] and Liuzijue [*MD* = −0.28 (95% CI: −0.64–0.08)].

### Punlication bias

The results of the publication bias analysis, including the funnel plot and corresponding statistical tests, are shown in Supplementary Appendix 1.

## Discussion

This systematic review and meta-analysis synthesized evidence from 22 RCTs (22–43) involving 1,871 patients to evaluate the effects of Traditional Chinese Medicine Breathing Exercises (TCMBEs), including Tai Chi, Baduanjin, Liuzijue, and Yijinjing, on pulmonary function, physical performance, and HRQoL in individuals with COPD. Aggregating all the results showed that there were statistically and clinically significant improvements in many areas of PR. TCMBE participation led to an increase in FEV1 of 0.20L, FVC of 0.22L, FEV_1_/FVC of 3.4%, FEV1% predicted of 4.98%, and 6MWD of about 42m, as well as a decrease in SGRQ by 13.76 points, CAT by 2.62 points, and mMRC by 0.50 points. This shows that mind-body activities do improve lung mechanics, exercise tolerance, and symptoms compared to just regular care or normal PR so it proves they work for treating COPD.

The results of this study are close to the ones from other studies which showed that TCMBEs improve physical as well as mental health. Tai Chi greatly improved 6MWD and SGRQ scores for people who had stable COPD (15); comparable improvements in exercise tolerance were noted with Tai Chi relative to walking or regular training (9); and Baduanjin resulted in beneficial alterations in FEV_1_, FEV_1_/FVC, and overall QoL (14). And then they also added to it with direct comparisons of various exercises. In the subgroup analysis, we can see that the improvement of spirometry index was most obvious for Liuzijue and Yijinjing (FEV1/FVC), Tai Chi improved most in terms of endurance, and Yijinjing experienced the biggest reduction in symptoms as well as feeling of breathlessness. This makes some TCMBE styles result in different physical and mental things happening. And they're all important and useful as well. An average improvement of 42m on 6MWD which is above the recognized minimal clinically meaningful threshold for COPD and equivalent to supervised aerobic training (6, 7). None of them had any negative effects, which means TCMBEs are safe to use and it's okay to take them for a long time, especially if you're old or out of shape.

Although TCMBEs share common features (slow movement, breath awareness, and attentional focus), each modality delivers a different mix of respiratory-specific training and locomotor demand, which may yield different outcome profiles. Liuzijue places a strong emphasis on paced breathing and prolonged expiration with phonation. This respiratory pattern may enhance expiratory control and breathing efficiency, potentially reducing air trapping and improving spirometric indices in COPD. Yijinjing incorporates deliberate stretching and trunk movements that may increase thoracic mobility and facilitate more comfortable ventilation; combined with its meditative pace, it may also influence symptom perception and health status scores. In contrast, Tai Chi involves continuous stepping, weight transfer, and coordinated whole-body movement, providing a more pronounced neuromotor and aerobic stimulus that may translate more directly into improved walking endurance (6MWD). Nevertheless, these interpretations should be viewed as hypothesis-generating because the number of trials differed across modalities and few RCTs measured intermediate physiological markers (e.g., respiratory muscle strength, dynamic hyperinflation, autonomic indices).

Multiple mechanisms likely elucidate these findings. Biomechanically, TCMBEs prioritize regulated diaphragmatic respiration and trunk expansion, enhancing ventilatory capacity, activating underutilized alveoli, and fortifying inspiratory and expiratory musculature.Liuzijue makes use of breath control by means of six sounds so as to permit more extended and robust exhalations, thereby fortifying the respiratory muscles and enhancing the clearing of the airways. Baduanjin and Yijinjing have symmetrical upper body stretches and gentle dynamic trunk rotations which can increase thoracic compliance and chest wall movement, resulting in better lung elasticity and breathing (11, 44, 45). Autonomicand inflammatory modulation maybe involved too. Slow, regular breathing increases vagal activity and decreases sympathetic outflow, which improves baroreflex sensitivity and lowers resting respiratory rat (a marker for less breathlessness) (46). Physiologically speaking, mind-body therapies decrease systemic inflammation markers such as interleukin-6 and tumor necrosis factor alpha, and increase antioxidant levels (47, 48). Neuroimaging and psychophysiological studies show that contemplative movement engages prefrontal cortices associated with attention control and mood regulation (19), And it's all about those psychosomatic pathways toward a better QoL. From this analysis we can see that there has been a decrease in the CAT and mMRC score, with a larger decrease seen in the yijinjing sub-group which could suggest there is some sort of synergy between respiratory retraining and building up emotional resilience. Slow, controlled breathing is associated with increased vagal modulation and reduced sympathetic arousal, reflected in improvements in HRV and baroreflex sensitivity. Although TCMBEs share a common mind–body framework, the “dose” of autonomic stimulation may differ across modalities because breathing is trained differently (e.g., paced diaphragmatic breathing vs. prolonged expiratory patterns with phonation), and because the movement component varies in rhythm and metabolic demand. For instance, Liuzijue explicitly trains prolonged expiration with sound production, which may facilitate slower respiratory frequency and stronger respiratory–cardiac coupling. Tai Chi combines breath awareness with continuous rhythmic movement and may influence autonomic balance through both paced breathing and moderate physical activity. However, most COPD RCTs have not measured HRV or baroreflex sensitivity directly; therefore, modality-specific claims regarding parasympathetic effects should be interpreted as theoretical rather than evidence-confirmed. Future trials should include standardized autonomic markers (e.g., HRV indices, baroreflex testing) and report breathing rate and exercise intensity to enable stronger mechanistic inference.

Compared to other kinds of PR methods such as walking on a treadmill or doing resistance exercises, TCMBEs give equal boosts to your ability to do things without needing as much work from your heart. These workouts are self-led and focus mainly on how well we move so that it's easier to breathe and harder to get exhausted, so people keep going (6, 9). In our meta-analysis the average change of 0.2L or 4.98% predicted FEV1 for improvement in FEV1 is consistent with center based endurance training (7), shows that if we don't have anyone to supervise our rehabilitation program, then maybe we could try using our minds and bodies instead. That this review got different outcomes from various exercises provides us clues concerning how to carry out certain rehab exercises better. Liuzijue's big changes to FEV1 and FVC fit nicely with its focus on taking longer breaths out, because that makes more expiratory reserve volume and strengthens control over how much air leaves your lungs when you breathe out. Tai Chi has better 6MWD improvement results because it contains balance training, rhythmic movement, and moderate aerobic exercise, which can improve locomotion endurance. Yijinjing had the biggest decrease in CAT score (-8.6 points) which showed that there were improvements in their psychological well being and difficulty breathing as a result of the stretching and meditation breathing techniques used in Yijinjing. The comparable improvement in the FEV1/FVC ratio with all modalities backs up the idea that the mechanical and autonomic effects of controlled breaths have equivalent impact no matter what form it takes.

Conventional pulmonary rehabilitation is typically built around aerobic and resistance training delivered at moderate intensity with progressive overload, aiming to improve peripheral muscle function, exercise tolerance, and ventilatory efficiency. By contrast, TCMBEs (e.g., Tai Chi, Baduanjin, Liuzijue, Yijinjing) combine controlled breathing, low-to-moderate intensity rhythmic movements, postural alignment, and mindful attention. This distinction suggests partially different mechanisms: traditional PR may exert its main effects through conditioning of the cardiopulmonary and musculoskeletal systems, whereas TCMBEs may additionally act via breathing pattern retraining (e.g., prolonged exhalation and diaphragmatic engagement), autonomic modulation, balance/coordination training, and improved symptom self-management. These features may help explain why some TCMBEs show consistent benefits on dyspnea- and HRQoL-related outcomes and can be delivered with minimal equipment, potentially supporting long-term maintenance after formal PR programs.

In practical terms, incorporating TCMBEs into PR offers distinct advantages. They are inexpensive, require little to no equipment, can be performed individually either in public spaces or at home, and are well suited for unsupervised maintenance practice. And these features cut down on big obstacles that stop people from sticking with it, especially when there aren't many official rehab treatments around (1, 3). They fit nicely alongside current pharmaceutics and so they make fine supplements for managing symptoms and improving general health. So policymakers and doctors should add structured TCMBE lessons, taught by good teachers, to outpatient and community recovery plans. But we have to be careful when interpreting the combined results as there is quite a bit of variation. It might be due to the fact that various individuals carried out the workouts differently because they performed them at different times, with varying levels of energy, and for different durations of time. Also, about 75% of the studies consisted of Asian populations, so there could be problems with culture and publishing bias, but they all followed Cochrane risk-of-bias criteria. Neither the subject nor the teacher could be blinded, so there might have been some effect of expectations, but this was reduced by objective spirometry results. And also there were not many trials which followed up for more than 6 months, so we do not know how long these results last. We need to do more research to find out whether keeping up with TCMBE treatment reduces flare-ups, hospital visits, and deaths over a long time.A lot of the papers did not give enough information about the bodies' own markers or the special pictures from the machines, so it was hard to understand how the special hats helped people breathe better.

A key limitation of this review is the substantial heterogeneity across the included RCTs. Variability existed in (1) intervention modality (Tai Chi, Baduanjin, Liuzijue, Yijinjing, or combined programs), (2) intervention “dose” and delivery, and (3) comparator intensity (usual care/education vs. conventional PR or other active exercise). Across trials, TCMBE frequency ranged from approximately 2 to 7 sessions per week, session duration ranged from 15 to 60 min, and total intervention length ranged from 4 weeks to 6 months. In addition, supervision differed (center-based classes, home-based practice with guidance, or mixed formats), and adherence reporting was inconsistent. These differences likely contributed to the high statistical heterogeneity observed in some pooled outcomes (e.g., FEV_1_, 6MWD, SGRQ), and they may partly explain why effect sizes differed across modalities. Furthermore, evidence was unevenly distributed across modalities. Tai Chi and Liuzijue were supported by more trials, whereas Yijinjing and mixed protocols were evaluated in relatively few studies. As a result, modality-specific estimates—especially for less-studied interventions—may be imprecise and sensitive to individual trials. Therefore, subgroup findings should be interpreted as exploratory rather than definitive. Future trials should report TCMBE protocols using standardized FITT descriptors (frequency, intensity, time, and type), include objective monitoring of exercise intensity where feasible, and apply longer follow-up to determine whether improvements translate into sustained benefits and fewer exacerbations or hospitalizations.

## Conclusion

This meta-analysis demonstrates that TCMBEs can enhance pulmonary function, increase exercise tolerance, and improve QoL in individuals with COPD. The integration of controlled breathing, gentle movement, and mindful focus appears to exert complementary rehabilitative effects that augment conventional pharmacologic and exercise-based interventions. Among the different approaches, Liuzijue and Yijinjing showed the greatest benefits for lung function and symptom relief, whereas Tai Chi was most effective in improving exercise endurance. Given their safety, affordability, and adaptability, TCMBEs should be incorporated into PR frameworks as evidence-based components of COPD management. Future large, well-designed RCTs with long-term follow-up are warranted to optimize dosage, clarify underlying mechanisms, and support global guideline integration.

## Data Availability

The original contributions presented in the study are included in the article/Supplementary material, further inquiries can be directed to the corresponding author.
