# Supplementary material for: The effects of traditional Chinese mind-body exercise on pulmonary rehabilitation in patients with chronic obstructive pulmonary disease: a systematic review and meta-analysis
Source: Front Med (Lausanne). 2026 Feb 11;13:1740693. doi: 10.3389/fmed.2026.1740693 (PMC12933424; doi:10.3389/fmed.2026.1740693)
Supplement: Supplementary file 1 [file Supplementary_file_1.docx]

Appendix 1

The funnel plot was constructed with the mean difference as the horizontal axis and the standard error as the vertical axis. Ideally, in the absence of publication bias or small-study effects, the data points should be symmetrically distributed on both sides of the plot, forming an inverted funnel shape. As shown in the figure S1, most studies are clustered in the middle-upper area of the plot, with a slight concentration on the right side and relatively fewer points on the left. This asymmetry may suggest the presence of a mild publication bias, indicating that studies with positive results are more likely to be published. Furthermore, the lower part of the plot contains fewer studies with larger standard errors, implying that small-sample or non-significant studies may be underrepresented. Overall, the plot suggests a slight possibility of publication bias or small-sample bias. However, the degree of asymmetry is not substantial and should be further assessed using Egger’s or Begg’s tests.


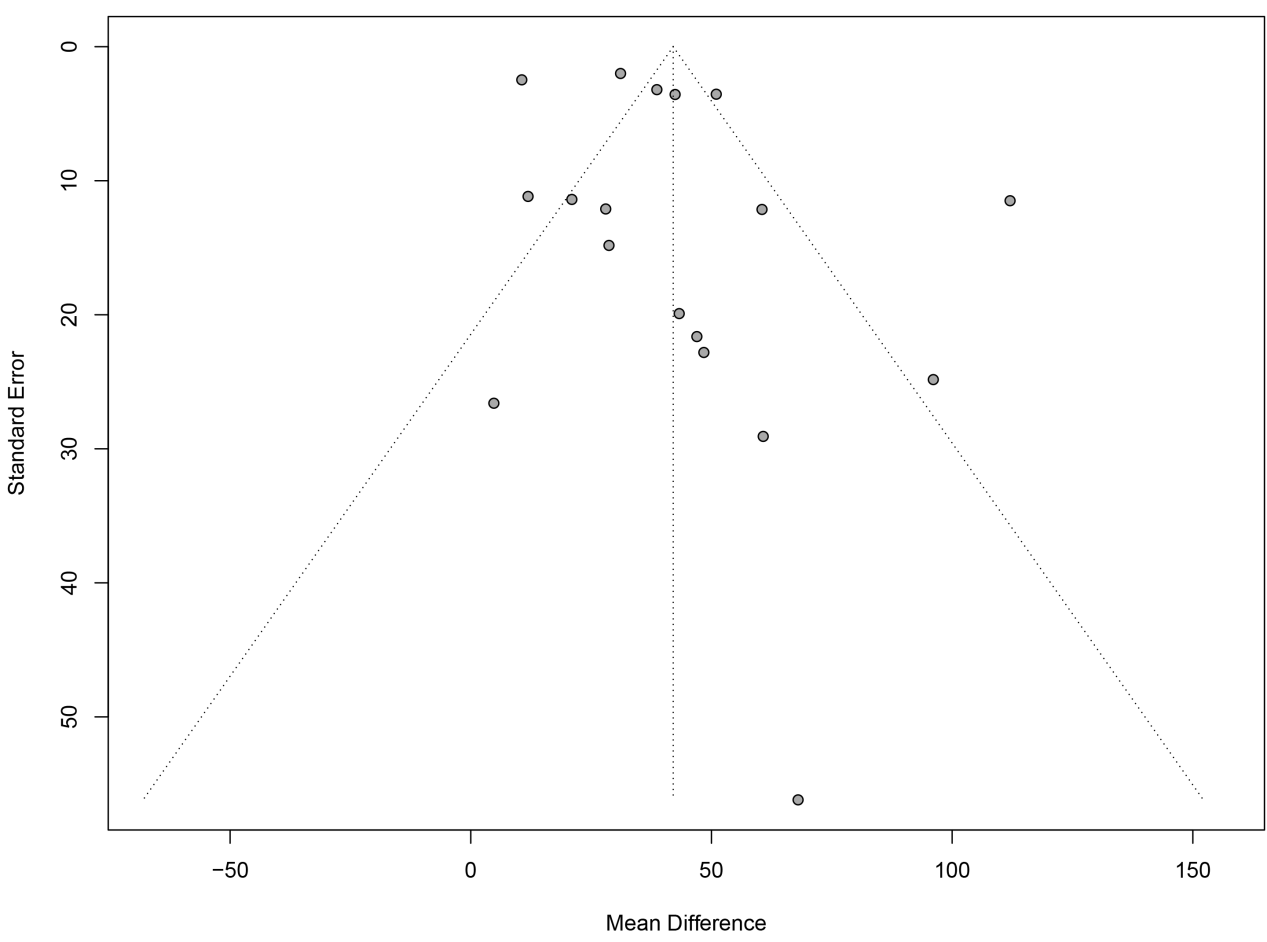
、

Figure S1. Funnel plot of mean differences in 6‑minute walk distance (6MWD)
